# Feasibility of Nurse-Led Advance Care Planning Before Pre-cardiac Procedures: A Descriptive Study

**DOI:** 10.7759/cureus.48347

**Published:** 2023-11-06

**Authors:** Kyoko Shigetomi, Eiji Hiraoka, Miho Takahashi, Tadanori Nabeshima, Yasuhiro Norisue, Kotaro Obunai, Joji Ito, Minoru Tabata

**Affiliations:** 1 Department of Cardiovascular Surgery, Tokyo Bay Urayasu Ichikawa Medical Center, Chiba, JPN; 2 Department of Internal Medicine, Tokyo Bay Urayasu Ichikawa Medical Center, Chiba, JPN; 3 Department of Critical Care Medicine, Tokyo Bay Urayasu Ichikawa Medical Center, Chiba, JPN; 4 Department of Cardiology, Tokyo Bay Urayasu Ichikawa Medical Center, Chiba, JPN; 5 Department of Cardiovascular Surgery, Juntendo University Graduate School of Medicine, Tokyo, JPN

**Keywords:** nurse-led acp, advance care planning (acp), shared decision-making, high-risk cardiac surgery, goal of care

## Abstract

Background

Shared decision-making is important for deciding whether to perform surgery, especially high-risk surgery, or end-of-life care in cases of serious complications after the surgery. In shared decision-making, surgeons should be aware of patients’ values. Therefore, advance care planning (ACP) before the surgery is important. In Japan, the feasibility of ACP, particularly preoperative nurse-led ACP, is yet to be evaluated.

Methodology

This retrospective, single-center, descriptive study included all adult candidates for open-heart or thoracic aortic surgery and transcutaneous aortic valve implantation (TAVI) referred by their surgeon for a nurse-led preoperative ACP between April 1, 2020 and December 31, 2021. The nurse conducted semi-structured interviews with patients regarding goals of care, unacceptable conditions, undesired procedures, advance directives, and their surrogates and documented them. The content of these interviews and their influence on decision-making were retrospectively investigated.

Results

Sixty-four patients (median age, 82 years; Society of Thoracic Surgeons (STS) score, 7.9; EuroSCORE II, 4.2; JapanSCORE, 7.0) were included (open-heart or thoracic aortic surgery 24, TAVI 40). Among them, 63 (98.4%), 56 (87.5%), and 13 (20.3%) patients articulated their goals of care, unacceptable conditions, and undesired procedures. Only one (1.6%) had a written advance directive. Although all of the patients could designated their surrogate, only 11 (17.2%) had shared their values disclosed in the pre-procedure ACP communication with their surrogates. Two patients who planned to undergo open-heart surgery disclosed their wish not to undergo the surgery only to the nurses but could not tell their surgeon; thereafter, the surgery was canceled. Three patients died after the procedure; however, the patients’ value disclosed in ACP was not used for the end-of-life decision.

Conclusion

Nurse-led ACP can be implemented before high-risk cardiac procedures. It may have an impact on the decision-making of surgery although the ACP content may not be utilized for the end-of-life discussion after the procedures between surgeons and the family member.

## Introduction

Complications of cardiac surgery can be serious and include death, mediastinitis, acute renal failure, and stroke. Transcutaneous procedures, such as transcutaneous aortic valve implantation (TAVI), were developed to reduce periprocedural invasiveness and complications. Nonetheless, complications can occur, including death (1.7%), disabling stroke (1.7%), serious bleeding (5.8%), and pacemaker placement (8.5%) [1]. Serious complications can result in a significant decreases in physical and cognitive functions. Some patients live in chronic critical condition after intensive care treatment [2]. Therefore, shared decision-making regarding whether to perform such high-risk procedures is important. For shared-decision-making, it is important to understand the patients’ values and goals of care before surgery. Among people who died between 2000 and 2006 in the USA, 42.5% needed decision-making about treatment in the final days of life. Of them, 70.3% lacked decision-making capacity [3]. Furthermore, a systematic review showed that surrogates predict patients' treatment preferences with 68% accuracy [4].

In Japan, the Ministry of Health, Labor and Welfare established guidelines for the decision-making process for end-of-life care in 2007, which were updated in 2015 and 2018 [5]. The guidelines emphasize the importance of not only patients’ medical conditions but also their values and preferences in the decision-making process in end-of-life care [5]. Therefore, advance care planning (ACP) is strongly recommended [5]. The Japanese Circulation Society (JCS) also recommends ACP for heart failure patients in its 2018 clinical guidelines of heart failure [6]. Surgical guidelines in the USA recommend ACP before high-risk geriatric surgery [7,8]. The JCS 2022 perioperative management clinical guideline also emphasizes the importance of ACP before high-risk surgeries [9]. However, the feasibility in Japan has not yet been investigated. Furthermore, although nurse-led ACP is reportedly feasible for medical conditions [10], it has not yet been evaluated for high-risk cardiac procedures. At our institution, nurse-led ACP before cardiac procedures was introduced in 2020 for high-risk patients. We retrospectively reviewed our single-center experience with nurse-led ACP before TAVI and open-heart surgery. We also evaluated its influence on decision-making regarding the cardiac procedures and life-prolonging treatment after the development of serious complications.

## Materials and methods

Ethical approval

The protocol for this retrospective descriptive study was approved by the Institutional Review Board of Tokyo Bay Urayasu Ichikawa Medical Center, Chiba, Japan (approval number 703). The requirement for informed consent from each patient was waived by the Ethics Committee because only retrospective, de-identified patient data were used.

Study design

This is a retrospective, single-center, descriptive study.

Subjects

This study included all adult patients who were candidates for open-heart or thoracic aortic surgeries and TAVI and were referred by their surgeon for nurse-led preoperative ACP between April 1, 2020, and December 31, 2021.

Setting

Tokyo Bay Urayasu Ichikawa Medical Center is located in an urban city near Tokyo and has a heart center where various high-risk cardiac procedures, including TAVI, and open-heart and aortic surgery, are performed. Palliative care teams, cardiologists, and cardiac surgeons discussed preoperative ACP, including elements and interview scripts (Table 1), and the importance of shared decision-making since April 2019. It was implemented on April 1, 2020. The main purpose of the preoperative ACP was to facilitate shared decision-making regarding whether to undergo a high-risk procedure and life-prolonging treatment in the case of a critical condition regardless of whether it was related to the procedure. When the surgeon considered the surgical risk to be high, e.g., the predicted mortality >3% based on the Society of Thoracic Surgeons (STS) score or EuroSCORE II, they referred them to nurse-driven ACP. Of note, the nurses were well trained in ACP communication and had a wealth of experience in ACP communication with acute care patients admitted in internal medicine services.

**Table 1:** Elements and semi-structured scripts for preprocedural advance care planning

Element	Semi-structured script for preprocedural advance care planning
1. Goal of care	a) What makes your life meaningful? What is your quality of life? b) What are you hoping to achieve by getting a surgery? What are things you look forward to doing after getting your surgery?
2. Unacceptable condition	Regardless of whether you undergo the procedure, you may have severe illness, such as stroke, heart attack, or severe infection. a) What if things do not go well? b) These complications can cause significant disability and keep you from achieving your goals. What is the condition you would find unacceptable? Would you consider that death is better than that condition? For instance, what would you think of living with artificial nutrition due to coma? Would this change if these were permanent states?
3. Undesired procedures	Are there any medical procedures you never want to receive, such as cardiopulmonary resuscitation, tracheostomy, percutaneous gastric tube placement, or open surgery?
4. Written form of advance directive	Do you have written form of advance directive?
5. Surrogate decision-maker	Have you designated a surrogate decision-maker?
6. Sharing your value and preference with surrogate	Have you discussed the above your values and preferences with your surrogate?

The ACP nurses interviewed patients before the surgery about their values and treatment preferences using a semi-structured interview script to elucidate their values, as explained in the ACP element. The findings were documented in the patients’ charts and shared with the referring surgeons, who were expected to utilize them for shared decision-making in undergoing the index surgery and for life-prolonging treatment in cases of critical conditions in the future, including serious surgical complications. This project was led by a palliative care team, consisting with physicians and nurses.

Notably, they were supposed not to explain the individual medical condition and individual surgical procedure, including its complication, which the surgeon and nurse practitioner specialists were supposed to explain to the patient. The main role of ACP nurses was to discuss the patients’ value. They were allowed to inform the patients that the surgery or their disease itself might possibly be complicated with serious conditions, such as stroke, infection, or heart attack, which may result in physical and mental dysfunction as a general information. 

Elements of ACP

We reviewed the ACP elements and interview scripts used in previous studies to disclose patient values [11,12]. Then, we discussed which elements and interview script would help patients and surgeons in decision-making regarding the procedure and would help the surrogate in decision-making when patients became critical and lost decision-making capacity in the future and modified them. Finally, we included six elements: 1) goal of care, 2) unacceptable conditions, 3) undesired procedures, 4) presence of a written form of advance directive), 5) designated surrogate, and 6) sharing these values and treatment preferences with the surrogates. We performed ACP with 10 patients as a pilot study, received their feedback, and modified the interview script to the final version shown in Table 1.

Data collection

The patient characteristics were obtained through a retrospective chart review. The following information was obtained: age, sex, Katz index, clinical frailty scale for physical functional status, STS score, EuroSCORE II, and JapanSCORE. A history of diabetes mellitus, hypertension, chronic kidney disease, maintenance dialysis, stroke, and cognitive disorders was also obtained. The percentage of patients who articulated each element was also calculated, and a retrospective chart review was conducted to assess whether these were utilized for decision-making regarding the procedure. In cases of mortality during index hospitalization, data on whether the surgeon utilized patients’ values disclosed in the ACP for decision-making of end-of-life care before they died were collected by a chart review.

Statistical analysis

Continuous variables were expressed as medians (interquartile range (IQR): 25% and 75%), and discrete variables were summarized as percentages. All statistical analyses were conducted using IBM SPSS Statistics for Windows, version 22 (released 2013; IBM Corp., Armonk, New York, United States).

## Results

Patients’ characteristics

A total of 64 patients were included in the study. Of these, 40 and 24 planned to undergo TAVI and open-heart or thoracic aortic surgery, respectively. TAVI and open-heart or thoracic aortic surgery were performed in 185 and 105 cases (290 cases in total), respectively, during the same period, and nurse-led ACP was performed in approximately 22% (64/290) of the patients. The baseline characteristics, in-hospital mortality, and final decision to undergo the procedure are shown in Table 2. The median (IQR) age was 82 (76-87) years, and 28 patients were male (43.8%). The cardiac surgical risk was calculated as follows: STS score 7.9 (5.0, 13.1), EuroSCORE II 4.2 (3.1, 6.0), and JapanSCORE 7.0 (3.8, 9.9). The in-hospital death rate was 4.7% (3/64).

**Table 2:** Patient characteristics DM: diabetes mellitus, IQR: interquartile range *Katz index: assessment of patients’ functional capacity in activity of daily livings with range 0 to 6; 0=very dependent, 6=independent. **Clinical frailty scale: range 1 to 9, 1=very fit, 2=fit, 3=managing well, 4=living with very mild frailty, 5=living with mild frailty, 6=living with moderate frailty, 7=living with severe frailty, 8=living with very severe frailty, 9=terminally ill.

	All (N=64)	Transcatheter aortic valve implantation (N=40)	Open-heart and thoracic aortic surgery (N=24)
Age (years), median (IQR)	82 (76, 87)	83 (79, 87)	81 (76, 87)
Male sex, n (%)	28 (43.8)	24 (60.0)	4 (16.7)
STS score, median (IQR)	7.9 (5.0, 13.1)	8.0 (5.3, 13.2)	7.4 (1.9, 12.2)
EuroSCORE II, median (IQR)	4.2 (3.1, 6.0)	4.4 (3.3, 6.8)	3.4 (2.0, 6.0)
JapanSCORE, median (IQR)	7.0 (3.8, 9.9)	7.6 (4.3, 10.2)	6.0 (1.8, 9.0)
DM, n (%)	23 (35.9)	18 (45.0)	5 (20.8)
Hypertension, n (%)	42 (65.6)	25 (62.5)	17 (70.8)
Chronic kidney disease, n (%)	28 (43.8)	14 (35.0)	14 (58.3)
Hemodialysis, n (%)	14 (21.9)	14 (35.0)	0
Stroke, n (%)	12 (18.8)	9 (22.5)	3 (12.5)
Malignancy within 5 years, n (%)	3 (4.7)	3 (7.5)	0
Cognitive disorder, n (%)	5 (7.8)	4 (10.0)	1 (4.2)
Katz index*, median (IQR)	6 (6,6)	6 (6,6)	6 (6,6)
Clinical frailty scale**, median (IQR)	4 (3, 5)	4 (3, 5)	3 (2.25, 4.75)
In-hospital death, n (%)	3 (4.7)	2 (5.0)	1 (4.2)
Final decision: not undergo procedure, n (%)	3 (4.7)	0 (0)	3 (12.5)

Patients’ value

All the patients participated in preoperative ACP interviews. As shown in Table 3, almost all the patients (98.4%) articulated the goal of care. Up to 87.5% of the patients articulated unacceptable conditions in which they would die rather than prolong their life: 32.8%, living unconscious with artificial nutrition; 64.1%, total dependence; and 29.7%, being unable to communicate with their family. Only 20.3% of the patients articulated undesired procedures. Of note, in terms of procedures, the patients would not desire to do in any situation; two out of 24 (8.3%) patients who were scheduled for open surgery confessed to the nurses that they honestly did not want open surgery in any situation. They said that they could not express their wishes to avoid undergoing the procedure to their surgeons by themselves. Furthermore, five out of 40 (12.5%) patients in the TAVI group reported that they did not want to undergo open-heart surgery, even when they needed to switch to open-heart surgery to survive because serious complications occurred during the procedure. Although all patients were able to designate their surrogates, only 17.2% (11 out of 64) had shared their values (goal of care, unacceptable condition, and undesirable procedure) with their designated surrogates. Only 1.6% of the participants (one out of 64) had written advance directives.

**Table 3:** Patients’ responses to presurgical advance care planning Data are expressed in n (%). PEG: percutaneous endoscopic gastrostomy

	All (N=64)	Transcatheter aortic valve implantation (N=40)	Open heart and thoracic aortic surgery (N=24)
Articulated goal of care	63 (98.4)	40 (100)	23 (95.8)
Articulated unacceptable health condition	56 (87.5)	32 (80.0)	24 (100)
Unconscious with artificial nutrition	21 (32.8)	10 (25.0)	11 (45.8)
Total dependence	41 (64.1)	25 (62.5)	16 (66.7)
Cannot communicate	19 (29.7)	10 (25.0)	9 (37.5)
Articulated undesired procedure	13 (20.3)	7 (17.5)	6 (25.0)
Open surgery	7 (10.9)	5 (12.5)	2 (8.3)
PEG	6 (9.4)	2 (5.0)	4 (16.7)
Tracheostomy	4 (6.3)	2 (5.0)	2 (8.3)
Cardiopulmonary resuscitation	0 (0)	0 (0)	0 (0)
Presence of advance directive	1 (1.6)	1 (2.5)	0 (0)
Designated surrogate	64 (100)	40 (100)	24 (100)
Having shared above with surrogate	11 (17.2)	7 (17.5)	4 (16.7)

Influence of the presurgical ACP on the surgery decision-making process

Among those planning open surgeries, two patients disclosed that they never wanted open surgery, which their surgeon did not know. After nurse-led ACP, surgeons discussed surgical options with patients and other treatment options. They decided to cancel the open surgery and administered only medical treatment to the two patients. Otherwise, this retrospective chart review could not disclose precise information about the influence of ACP on the decision-making of the index procedure.

Influence of the presurgical ACP in the decision-making process for end-of-life treatment

Three patients died during the index hospitalization. Intensive care was provided to all patients: 2, CPR; 3, mechanical ventilation with 1, tracheostomy; 1, new hemodialysis; and 2, mechanical circulatory support. In all cases, the family, instead of the patients, needed to make decisions regarding intensive care treatment because they lost their decision-making capacity. They decided to withdraw intensive care and implement comfort management only with do-not-resuscitate (DNR) decisions after discussion with the family when it was impossible to save their lives. According to the chart documentation, surgeons did not discuss the patients’ values and preferences expressed in the preoperative ACP at the time of decision-making regarding withdrawal of intensive care and DNR decisions before death (Table 4). 

**Table 4:** Clinical course of death cases M: male. F: female, TAVI: transcatheter aortic valve implantation, NOMI: non-obstructive mesenteric ischemia, PEG: percutaneous endoscopic gastrostomy, CHF: Y: yes, N: no, ACP: advance care planning, CPR: cardiopulmonary resuscitation, HD: hemodialysis, IABP: intra-aortic balloon pumping, PCPS: percutaneous cardiopulmonary support, 0: not performed, 1: performed

Age	Sex	Index procedure	Cause of death	ACP: Unacceptable condition articulated	ACP: Undesirable procedure for end of life articulated	Cardiopulmonary resuscitation	New HD	Mechanical ventilation	Mechanical circulatory support	Decision of withhold or withdraw of life prolonging treatment	Utilization of presurgical ACP for end-of-life decision
87	F	Open- heart surgery	NOMI, heart failure after the procedure	Y:	Y PEG Tracheostomy	0	0	1	0	Y	N
82	F	TAVI	Sepsis, bleeding after the procedure	Y:	N	1	1	1 (Tracheostomy)	1 (IABP, PCPS)	Y	N
75	M	TAVI	Sudden death before TAVI	Y:	N	1	0	1	1 (PCPS)	Y	N

## Discussion

We report that nurse-led ACP could be implemented before high-risk cardiothoracic procedures, either catheter-based or surgical, under consultation by surgeons. In some cases, ACP interviews by the nurse influenced the decision-making to not undergo surgery.

A survey conducted by the Japanese Ministry of Health, Labour and Welfare in 2013 found that 70% of respondents would not like to undergo invasive treatment if they became terminally ill, and 70% would like to establish advance directives, although only 3% of them did [13]. In another survey of middle-aged and older adults in Tokyo, Japan, 60% of the respondents stated that they would like to express their wishes regarding advance directives, but <10% had already done so [14]. No studies have reported patient responses to ACP before high-risk cardiac procedures or how they are actually used in decision-making in Japan. To the best of our knowledge, this is the first study to evaluate this aspect in Japan.

Our study showed that up to 98.4% of the patients articulated the goal of care by answering the following questions: What are you hoping to achieve by undergoing surgery? What things do you look forward to doing after surgery? In shared decision-making, if their expected goal cannot be achieved, physicians need to inform patients of it and inform them of what goal is achievable by the surgery. If such a corrected goal is not acceptable to patients, they should be advised not to undergo surgery [15].

Our report showed that 87.5% of the patients articulated an “unacceptable condition” in which they would withdraw or withhold life-prolonging treatment and die. Only 17.2% had shared this value with their surrogates at the time of ACP interviews. Therefore, presurgical ACP is potentially useful for their surrogate to know the patient 's value.

ACP interventions reportedly increased the occurrence of end-of-life care discussion, concordance between preferences for care and delivered care, and improved decisional conflict of end-of-life care in non-surgical settings [16,17,18]. It was also reported to improve patients’ satisfaction with healthcare in non-perioperative settings [16,17,18]. The effects of preoperative ACP have also been investigated in patients who underwent surgery. Concordance between patients and their surrogates in terms of postoperative use of life-sustaining treatment preferences was improved by ACP through nurse-led interviews before cardiac surgery, and decisional conflict was decreased in a clinical scenario-based questionnaire [19,20]. The feasibility of preoperative ACP communication before high-risk cardiac surgeries has also been demonstrated in the USA [12]. However, no research has investigated the translation of this preoperative ACP communication into real practice, including decisional conflict, anxiety, satisfaction with decision-making in the index surgery, or regret regarding the decision to undergo the surgery among patients and their families [21,22]. Therefore, further studies are needed.

One systematic review showed that ACP implementation reduced the cost and utilization of medical resources, such as ICU admission, in non-perioperative settings [18]. One recent study showed consistent results [23]. However, no study has investigated that presurgical ACP reduced the healthcare cost or healthcare utilization. Hence, further study is necessary.

In our study, three patients died in the hospital. The surgeon decided to withdraw the intensive care treatment and proceed to comfort measurement only with DNR after discussion with the family not because the patient’s conditions were “worse than dying,” as they had previously indicated in the ACP communication, but because death was inevitable. Presurgical ACP was not utilized in the end-of-life discussions between the treating surgeons and their families. Two mechanisms have been hypothesized. First, this might be because patients went into critical conditions emergently and their surgeons could not recognize the presence of ACP, and they could not utilize them for end-of-life decisions. Second, reportedly, some surgeons would not want to use a preoperative advance directive for critical conditions after surgery [24]. Another report demonstrated that surgeons are more reluctant to withdraw postoperative life-supporting therapy for patients with complications after surgery in the elective setting, irrespective of the patients’ values and treatment preferences [25]. In such cases, surgeons may continue life-prolonging treatment until the patients reach imminent demise [26], where death could not be prevented by any life-prolonging treatment. However, further studies are needed to investigate whether utilizing presurgical ACP helps patients’ and families’ decision-making to withdraw care or to decide DNR, even in imminent demise.

In this study, the ACP nurses were consulted by the surgeon and communicated with patients. However, who should perform the ACP communication is still debatable. Primary care physicians and specialists who treat life-limiting illnesses, such as oncologists and cardiologists, were considered proper candidates [10]. Nurses and social workers were also expected to become candidates [10]. One large randomized controlled trial (RCT) (Surfactant, Positive Pressure, and Oxygenation Randomized Trial (SUPPORT)) investigated the effects of nurse-led ACP [27]. Nurses had ACP communication with patients and informed their physicians of their patients’ goal of care and wishes for end-of-life care. However, this intervention did not improve any of the following outcomes: physician-patient communication about DNR preferences, incidence of DNR orders, physicians' knowledge of their patients ‘preferences not to be resuscitated, number of days spent in the ICU before death, receiving mechanical ventilation, or level of reported pain, which should be improved by ACP communication [27].

Meanwhile, ACP implementation by nurses or other trained healthcare workers improved end-of-care in one study [28]. In that study, the ACP intervention increased the number of patients whose end-of-life wishes were known and implemented accordingly by their treating physicians. It increased the number of patients involved in end-of-life care discussions. ACP decreases family psychological stress, anxiety, and depression. In the SUPPORT trial, although nurses informed the patients’ values to their primary care physicians, the final treating physicians might not possibly be informed of the patients’ value or might not have skill to utilize the information for shared decision-making of end-of-life care, possibly explaining the lack of benefit from nurse-led ACP. Whoever perform ACP communication with patients, if the treating physicians do not know the patient’s values or do not use it in the decision-making process, ACP could not work [29].

Limitations

We found that it was feasible to perform nurse-led preprocedural ACP under surgeon consultation. However, we were unable to investigate how feasible it is in Japan. Further prospective studies are needed to evaluate the following elements: how long it takes, how difficult it is for patients to answer the question before the procedure, how important the patients feel it is to disclose each value-related question for shared decision-making, and how comfortable they are with disclosing their values before surgery. We also need to investigate the feasibility from nurses’ perspective. We could not investigate its influence on patients' and their family’s decision-making regarding whether to perform the surgery, such as patient satisfaction, decisional conflict, and anxiety, and regret over their decision-making because of no comparison. This is the same case with the family’s decision-making regarding whether to perform life-prolonging treatment when the patients are in a critical condition and lose their decision-making capacity. Further studies are needed to investigate these.

## Conclusions

Nurse-led ACP could be implemented before high-risk cardiac procedures to disclose their values. It may influence their decision of the procedures although further studies are necessary to investigate the effect of nurse-led ACP on the decision of the procedure by comparing the presence of nurse-led ACP with the absence of it. Patients’ values disclosed in the presurgical ACP communication were not utilized for the decision making of end-of-life care for patients with imminent demise.
